# Introduction to Research Topic – Binocular Rivalry: A Gateway to Studying Consciousness

**DOI:** 10.3389/fnhum.2012.00263

**Published:** 2012-09-25

**Authors:** Alexander Maier, Theofanis I. Panagiotaropoulos, Naotsugu Tsuchiya, Georgios A. Keliris

**Affiliations:** ^1^Department of Psychology, Vanderbilt UniversityNashville, TN, USA; ^2^Department of Physiology of Cognitive Processes, Max Planck Institute for Biological CyberneticsTübingen, Germany; ^3^School of Psychology and Psychiatry, Monash UniversityClayton, Melbourne, VIC, Australia; ^4^Decoding and Controlling Brain Information, Japan Science and Technology AgencyChiyoda-ku, Tokyo, Japan; ^5^Computational Neuroscience Laboratories, Advanced Telecommunications Research Institute InternationalSeika-shi, Kyoto, Japan; ^6^Brain Science Institute, RIKENWako-shi, Saitama, Japan; ^7^Division of Biology, California Institute of TechnologyPasadena, CA, USA; ^8^Bernstein Center for Computational NeuroscienceTübingen, Germany

In 1593, Neapolitan polymath Giambattista della Porta publicly lamented that he was unable to improve his impressive productivity (he had published in areas as diverse as cryptography, hydraulics, pharmacology, optics, and classic fiction). Della Porta was trying to read two books simultaneously by placing both volumes side-by-side, and using each eye independently. To his great surprise, his setup allowed him to only read one book at a time. This discovery arguably marks the first written account of binocular rivalry (Wade, 2000) – a perceptual phenomenon that more than 400 years later still both serves to intrigue as well as to illuminate the limits of scientific knowledge. At first glance, binocular rivalry is an oddball. In every day vision, our eyes receive largely matching views of the world. The brain combines the two images into a cohesive scene, and concurrently, perception is stable. However, when showing two very different images (such as two different books) to each eye, the brain resolves the conflict by adopting a “diplomatic” strategy. Rather than mixing the views of the two eyes into an insensible visual percept, observers perceive a dynamically changing series of perceptual snapshots, with one eye’s view dominating for a few seconds before being replaced by its rival from the other eye. With prolonged viewing of a rivalrous stimulus, one inevitably experiences a sequence of subjective perceptual reversals, separated by random time intervals, and this process continues for as long as the sensory conflict is present.

This Frontiers Research Topic focuses on contemporary research on binocular rivalry and related visually multistable phenomena, covering a large variety of topics and techniques. It contains several reviews by leading experts in the field that provide perspectives on important insights that were gained during the past decades of research on rivalry, as well as a focus on outstanding conceptual, methodological, and empirical questions. Additionally, this collection includes research articles using psychophysical, computational, developmental, and imaging techniques that address fundamental questions related to the nature, origins, and neural implications of binocular rivalry. A short overview of the work is outlined in the following paragraphs (please refer to the original articles for further details).

Introducing a novel computational model based on a non-linear algorithm, Lehky (2011) suggests that – at least theoretically – each eye’s view can be extracted following binocular integration at later processing stages of the visual system, which could explain some apparent conflicts between previous psychophysical and neurophysiological results. An alternative model that employs an attractor-based neural network architecture previously used to understand working memory, attention, and decision making is presented by Theodoni et al. (2011). One of the hallmarks of binocular rivalry is its unpredictable switching between each eye’s views. Kang and Blake (2011) review our current understanding of these dynamic processes, and provide a new framework that integrates the empirical data. Single neuron recordings show perceptual modulation to binocular rivalry as early as V1 (Leopold and Logothetis, 1996; Keliris et al., 2010), ranging all the way to the frontal lobe (Panagiotaropoulos et al., 2012). The long-ranging effects of the neuronal processes that give rise to binocular rivalry can also be measured on the scalp electroencephalogram (EEG). Recent developments in this field, using a combination of new experimental and analytical approaches are reviewed by both Pitts and Britz (2011) as well as Kornmeier and Bach (2012), linking binocular rivalry to other multistable visual phenomena such as the famous “Necker Cube.” Wolf and Hochstein (2011) present evidence that binocular rivalry alternations can be modulated by high-level, semantic influences that might originate beyond the visual system. Paffen and Alais (2011) add to the discussion of high-level influences during binocular rivalry by reviewing the most recent literature on attentional influences on perceptual alternations, concluding that high-level selection processes can influence, but are not required to explain the temporal dynamics of binocular rivalry. Dieter and Tadin (2011) provide a complementary review of the interaction between selective attention and binocular rivalry, and place the results in a unifying framework that is based on the classic biased competition model (Desimone and Duncan, 1995). Focusing on low-level influences, on the other hand, Roumani and Moutoussis (2012) review literature on the role of visual adaptation for binocular rivalry alternations. While binocular rivalry is typically studied using artificial stimuli under laboratory conditions, Arnold (2011a,b) and O’Shea (2011) engage in a debate on how often binocular rivalry occurs under natural viewing conditions that ends in mutual agreement about the special nature of perceptual alternations. Alais and Parker (2012), on the other hand, demonstrate that even spatially matched images in each eye can rival for perceptual dominance, as long as their temporal modulation is sufficiently different. Andrews and Holmes (2011) revisit the question whether binocular rivalry is mutually exclusive with visual fusion between the two eyes, and present evidence that it is possible to extract stereoscopic depth from stimuli that are perceptually suppressed. Hudak et al. (2011) examine developmental differences of binocular rivalry by measuring its perceptual dynamics in pre-adolescent children. Fahle et al. (2011) combine tactile perceptual responses of subjects with measurements of pupil dilation to demonstrate that rather than constituting an all-or-none event; the internal decision about a perceptual transition seems to build slowly over time. Stuit et al. (2011) present data that suggests additive Gestalt-like grouping effects between binocular rivalry stimuli within and between hemispheres. In the same vein of testing visual interactions between binocular rivalry and other visual phenomena, Masuda et al. (2011) demonstrate that binocular rivalry suppression of key stimulus parts diminishes other visual illusions such as the Craik-O’Brien-Cornsweet effect. Rather than focusing on what is perceptually dominant during binocular rivalry, Zadbood et al. (2011) study what it takes to notice the physical removal of a stimulus that is perceptually suppressed. They find that the result depends on the specific visual feature under scrutiny (Zadbood et al., 2011), suggesting that two equivalent perceptual states can be accompanied by distinct neural events (see also Maier et al., 2007). Denison et al. (2011) tests the influence of perceptual history on binocular rivalry, and find that the visual system favors patterns for perceptual dominance if they are predicted by prior stimulation. Pelekanos et al. (2011) also find an effect of stimulus history that they liken to high-level modulation of perceptual selection. Stanley et al. (2011) review data on the initial (“onset”) stage of binocular rivalry that follows immediately after the presentation of binocular conflict. Their overview demonstrates that the initiation of binocular rivalry exhibits a variety of idiosyncratic properties that are absent during the ongoing perceptual fluctuations that follow (Stanley et al., 2011). Stienen and De Gelder (2011) present data that suggests that social cues such as fear expression can influence perceptual dominance during rivalry. Stein et al. (2011) investigate in the time it takes for visual stimuli to reach perceptual dominance under continuous flash suppression (Tsuchiya and Koch, 2005), a class of phenomena related to binocular rivalry. They caution against potential over-interpretation of the resulting data for inferences on the brain’s processing of unconscious stimuli (Stein et al., 2011). Genç et al. (2011) use diffusion tensor imaging (DTI) to demonstrate a direct relationship between anatomy of transcallosal connections and the perceptual dynamics of rivalry transitions. A refreshing new perspective gets provided by Miller et al. (2011), who suggest that some behavioral characteristics of the fruit fly resemble rivalry-like state changes in humans.

The reviews and empirical articles collected in this research topic demonstrate the many methodological and conceptual advances that have been made by the ever-growing field. The profound insights presented here do not only reflect on our understanding of binocular rivalry and its implications for visual awareness, perceptual organization, and binocular vision, but also have profound implications for our understanding of visual function in general.
